# The efficacy and safety of varenicline nasal spray for the management of dry eye signs: a systematic review and meta-analysis

**DOI:** 10.1186/s12886-023-03069-y

**Published:** 2023-07-14

**Authors:** Bader Bashrahil, Nada Taher, Ziyad Alzahrani, Ahmed Alnabihi, Abdulaziz Aldahlawi, Mohammed Alkhathlan, Saeed Alghamdi

**Affiliations:** 1 College of Medicine, King Saud bin Abdulaziz University for Health Sciences, Jeddah, Saudi Arabia; 2 King Abdullah International Medical Research Center, Jeddah, Saudi Arabia; 3 Department of Ophthalmology, Ministry of the National Guard-Health Affairs, Jeddah, Saudi Arabia

**Keywords:** Dry eye disease, Varenicline, Nasal spray

## Abstract

**Background:**

Dry eye disease (DED) is caused by a persistently unstable tear film leading to ocular discomfort and is treated mainly with tear supplementation. There is emerging evidence that nicotinic acetylcholine receptor (nAChR) agonists (e.g., varenicline and simpinicline) nasal sprays are effective for DED. Our systematic review and meta-analysis assessed the efficacy and safety of varenicline nasal spray (VNS) for DED treatment.

**Methods:**

The Medline, Embase, and Cochrane Central Register of Controlled Trials (CENTRAL) databases were searched. Only randomized controlled trials (RCTs) that evaluated the efficacy of VNS versus placebo were included. The efficacy endpoint was the mean change in the anesthetized Schirmer test score (STS), a measure of basal tear production, from baseline. The safety endpoints were serious adverse events (SAEs) and adverse events (AEs). The standardized mean difference (SMD) was used for continuous outcomes, while the risk ratio (RR) was used to demonstrate dichotomous variables. The certainty of the evidence was rated utilizing the Grading of Recommendations Assessment, Development, and Evaluation (GRADE) approach. The risk of bias assessment was conducted using the Revised Cochrane risk of bias tool for randomized trials.

**Results:**

Three RCTs (*n* = 1063) met the eligibility criteria. All RCTs had a low risk of bias. The meta-analysis found a statistically significant increase in the mean STS change from baseline on day 28. The pooled analysis found no significant difference between VNS and placebo in the frequency of SAEs and ocular AEs. However, VNS had a significant effect on developing nasal cavity-related AEs.

**Conclusion:**

VNS caused a highly significant improvement regarding the efficacy endpoint but caused an increased frequency of some nasal cavity-related AEs (i.e., cough and throat irritation). However, it caused neither SAEs nor ocular AEs. Included studies had a low risk of bias.

**Supplementary Information:**

The online version contains supplementary material available at 10.1186/s12886-023-03069-y.

## Background

Dry eye disease (DED), also known as keratoconjunctivitis sicca, is a disease of multifactorial etiology affecting one or more tear components leading to persistently unstable tear film with or without impaired characteristics. DED is often accompanied by irregular patterns of inflammation, neurosensory impairments, and ocular epitheliopathy, consequently causing abnormalities that would cause subjective visual dysfunction and ocular discomfort [1]. Worldwide prevalence estimates of DED reach up to fifty percent. Moreover, the incidence of DED escalates as age increases. Treatment options for DED are limited; therefore, novel interventions are emerging for DED management [1, 2].

Tear supplementation is the mainstay of DED management [2]. Other treatments, such as topical anti-inflammatory and immunosuppressive eye drops, are sparsely used [3]. Indefinite DED diagnosis and efficacy measures partially limit trials for DED drugs. Objective tests, such as tear break-up time (TBUT), anesthetized and non-anesthetized Schirmer test score (STS), and subjective questionnaires, such as the ocular surface discomfort index (OSDI), provide a moderate degree of diagnostic and prognostic value [2].

Artificial tear drops, the first line for most DED patients, have numerous limitations, such as requiring continuous instillation throughout the treatment to avoid relapse and build-up of DED signs and symptoms [3, 4]. Nicotinic acetylcholine receptor (nAChR) agonists are mainly used for smoking cessation as pills or patches; however, varenicline and simpinicline, two nAChR agonists, have been proposed as aqueous nasal sprays in emerging evidence, including high-quality randomized controlled trials (RCTs) [5–11]. The mechanism of action (MOA) is relatively new and was superficially dissected in limited evidence, but several articles proposed the same pathway [5, 7–9, 12, 13]. Varenicline nasal spray (VNS) exhibits high binding affinity for nAChR receptors and demonstrates partial and full agonist activity [7–9, 12–15]. It is postulated that VNS affects the trigeminal nerve ending within the anterior nasal cavity and activates the nasolacrimal reflux (NLR) [10, 13, 16]. NLR activation leads to increasing the production of tear films through the lacrimal functional unit (LFU) which consists of meibomian glands, lacrimal glands, and goblet cells that secrete componenets of tear films (mucin, aqueous, and lipid) [10, 12, 17, 18]. There is some evidence that the activation of the LFU via the trigeminal parasympathetic pathway (TPP) may improve DED in patients with Sjögren’s syndrome as a theraputic effect of oral muscarinic acteylcholine receptor agonists [19–22]. This, however, was associated with systemic adverse events (AEs) that pertained to their high systemic bioavailabitiy that are non-selective to LFU and DED pathphysiology [19–21]. Therefore, VNS was proposed as a selective option with a non-ocular site of action and was compared to an oral formulation with regards to pharmacokinetics and pharmacodynamics [13].

VNS is the first nasal spray approved by the United States Food and Drug Authority (FDA) as an intervention for DED (Tyravya™) [23]. Intranasal solution route of administration (ROA) is known as a cause of nasal cavity side effects. The indirect ROA to manage disease of ocular pathology raises uncertainty concerning the effectiveness and safety of such a unique intervention [6, 12]. As evidence of using VNS for DED is emerging, the topic was neither systematically reviewed nor sufficiently critically appraised.

In this article, a systematic review and meta-analysis were performed to evaluate the efficacy and safety of varenicline nasal spray for managing DED with different doses listed as subgroups against placebo. Patients were assessed for STS, serious adverse events (SAEs), and AEs.

## Methods

Our study was registered before the systematic search in PROSPERO (CRD42022343175) and reported this article according to the Preferred Reporting Items for Systematic Reviews and Meta-Analysis (PRISMA) checklist.

### Eligibility criteria

This study exclusively included studies that compared VNS to placebo and measured their treatment effect through STS. We excluded non-RCTs and studies that included patients with corneal, conjunctival, or other ocular cofounding conditions. We included studies in which participants had a prerequisite of baseline STS measurement at visit 1. The change in mean anesthetized STS from baseline to day 28, to estimate the basal tear production, was set to be our primary efficacy endpoint. Furthermore, we included the number of events of both SAEs and AEs to appraise the safety profile. Subsequently, AEs were divided into ocular and nasal cavity-related adverse events. VNS is prescribed in a multiple-dose gradient (0.12 mg/mL [low-dose], 0.6 mg/mL [mid-dose], and 1.2 mg/mL [high-dose]). However, the low-dose VNS was reported only in one RCT compared to mid-dose and high-dose. Consequently, our study evaluated mid-dose and high-dose only, and the outcomes mentioned above were described per dose.

### Search strategy

Our study systematically searched the Medline, Embase, and Cochrane Central Register of Controlled Trials (CENTRAL) databases from database initiation to July 6, 2022, without any restriction on date or language. References of the included RCTs were inspected for relevant RCTs that were missed during the systematic search process. The search strategy is provided in the Additional file 1.

### Study selection and data extraction

Independently and in pairs, two reviewers complied with the eligibility criteria and performed title and abstract screening, full-text assessment, and data extraction from the included reports. Discrepancies were discussed with a third reviewer or resolved through consensus before further advancement.

### Meta-analysis

Data analysis was performed using RevMan (Review Manager) version 5.3 (Cochrane Collaboration). The meta-analysis was performed using the random-effects model. A 95% confidence level and *P* < 0.05 were set for statistical significance. The statistical heterogeneity was assessed using the I^2^. We used the mean change in anesthetized STS from baseline on day 28 as the sole continuous variable, and the standardized mean difference (SMD) was used as the effect measure. Dichotomous outcomes (SAEs and AEs) were represented as risk ratios (RR) and pooled using inverse variance weighting. Subgroup analysis was performed by dividing the VNS arm into mid-dose and high-dose subgroups compared to the placebo intranasal spray.

### Certainty of evidence

The quality of evidence for each outcome was assessed using the Grading of Recommendations Assessment, Development, and Evaluation (GRADE) criteria [24]. The GRADE instrument, a Cochrane-recommended technique, was used to examine evidence quality and grading of recommendation strength in the included studies in the meta-analysis [25]. This evaluation considered factors such as research design, inconsistency, indirectness, heterogeneity, imprecision, publication bias, and other features stated by papers included in this systematic review. The quality of the evidence was then categorized as high, moderate, low, or very low [24, 25].

## Results

**Fig. 1:**
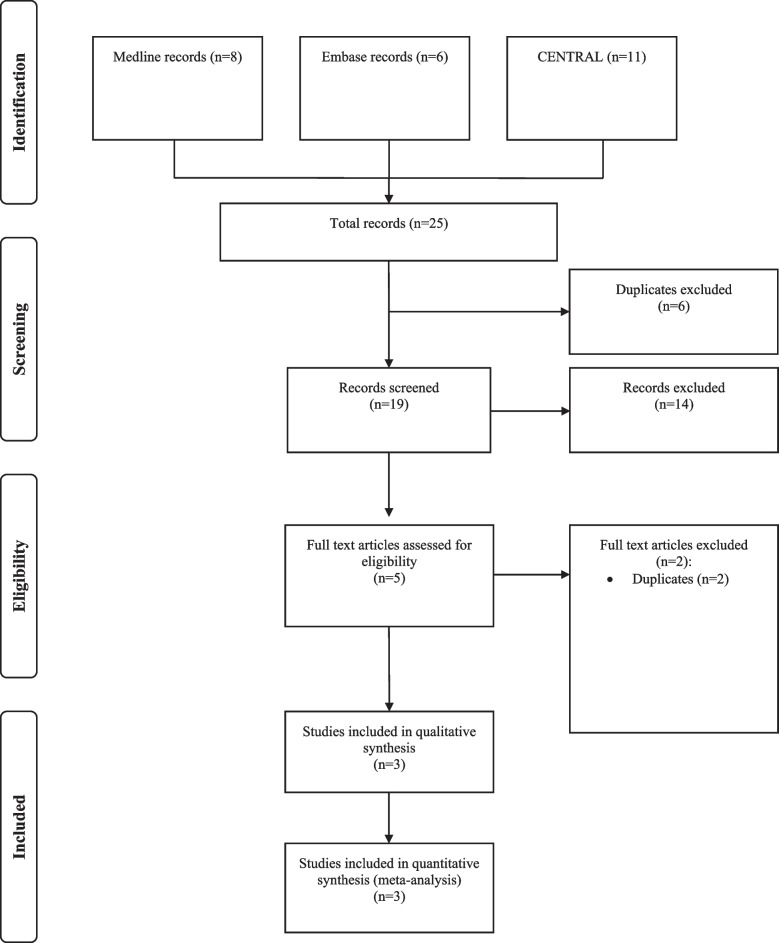
Study flow diagram. CENTRAL, Cochrane Central Register of Controlled Trial; RCT, randomized controlled trial

After the systematic search, 25 reports were identified, including six duplicates, resulting in 19 reports. Of the 19 reports, 14 were excluded due to unmatched eligibility. Eventually, five reports of 3 RCTs were included (Fig. 1).

### Trial characteristics

These trials yielded 1063 participants. Their mean age ranged from 51.4 to 67.4. Gender-wise, females comprised approximately 813 of these participants (76.5% of participants). Out of the 1063 participants, 241 were Latino or Hispanic, while the remainder were identified as of non-Latino or Hispanic ethnicity Table 1.

**Table 1:** Trial characteristics

Author, Journal, Study (Reference)	VNS dose (mg/mL)	Number of participants^a^		Number of participants^b^		Ethnicity		Gender	
		VNS	Placebo	VNS	Placebo	Latino or Hispanic	Not Latino or Hispanic	Male	Female
Wirta, Ophthalmology, ONSET-2 [8]	0.6	239	228	260	252	100	658	182	576
	1.2	212		246					
Hugo Quiroz-Mercado, The Ocular Surface, MYSTIC [9]	0.6	36	32	41	41	123	0	23	100
	1.2	29		41					
Wirta, Cornea, ONSET-1 [7]	0.12	47	43	47	43	18	164	45	137
	0.6	46		48					
	1.2	40		44					

^a^Number of participants at randomization

^b^Number of participants at study completion

### Risk of bias assessment

**Fig. 2:**
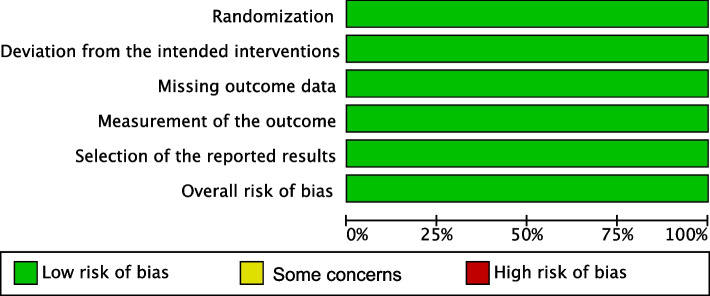
Risk of bias graph

**Fig. 3:**
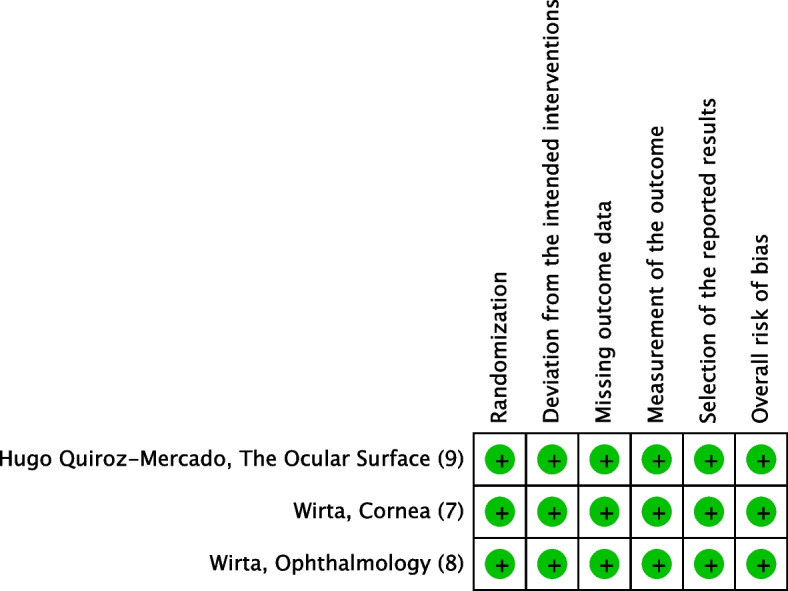
Risk of bias summary

Independently and together, two reviewers used the Revised Cochrane risk of bias tool to assess the risk of bias in the eligible RCTs. Each study's risk of bias was reviewed and scored as high, low, or some concerns. Discrepancies between the reviewers were resolved through discussion until an agreement was reached [26] (Fig. 2,3).

### Efficacy

**Fig. 4:**
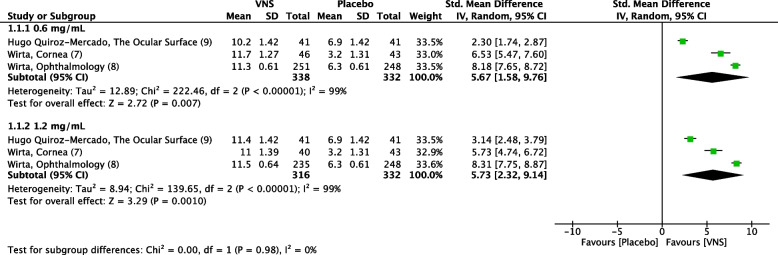
Forest plot of the mean change of Schirmer test score from baseline at day 28. CI, confidence interval; IV, inverse variance; SD, standard deviation; VNS, Varenicline nasal spray

**Fig. 5:**
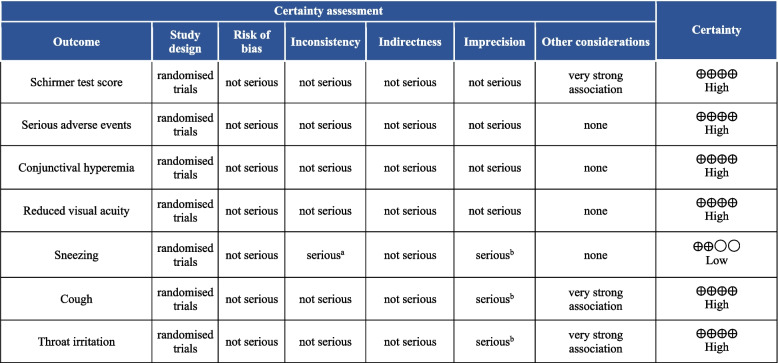
Grading of Recommendations Assessment, Development, and Evaluation (GRADE) evidence profile. **a** High heterogeneity. **b** Wide confidence interval

Three RCTs [7–9] reported STS as one of their efficacy outcomes. Both doses caused a substantial mean change of STS from baseline at day 28 versus the placebo nasal spray. Mid-dose showed almost an identical effect (SMD = 5.67 95% CI 1.58 – 9.76, *P* = 0.007, I^2^ = 99%) to high-dose (SMD = 5.73 95% CI 2.32 – 9.14, *P* = 0.0010, I^2^ = 99%). Nevertheless, no significant differences were found between mid-dose and high-dose subgroups (*P* = 0.98, I^2^ = 0%) (Fig. 4) (High quality of evidence) (Fig. 5).

### Serious adverse events

**Fig. 6:**
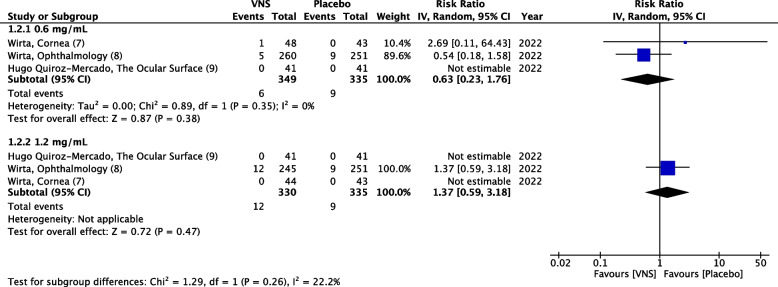
Forest plot of serious adverse events. CI, confidence interval; IV, inverse variance; SD, standard deviation; VNS, Varenicline nasal spray

SAEs were reported in three RCTs [7–9]. The pooled analysis assessed the incidence of SAEs from study initiation until the last follow-up visit. Mid-dose subgroup reported 6 SAEs in a sample of 349 patients (RR = 0.63, 95% CI 0.23–1.76, *P* = 0.38, I^2^ = 0%) and the high-dose subgroup reported 12 events in 330 patients (RR = 1.37, 95% CI 0.59–3.18, *P* = 0.47, I^2^ = not applicable). Similar to the placebo, both doses did not cause increased SAEs. No significant differences were found between doses (*P* = 0.26, I^2^ = 22%) (Fig. 6)(High quality of evidence) (Fig. 5).

### Ocular adverse events

**Fig. 7:**
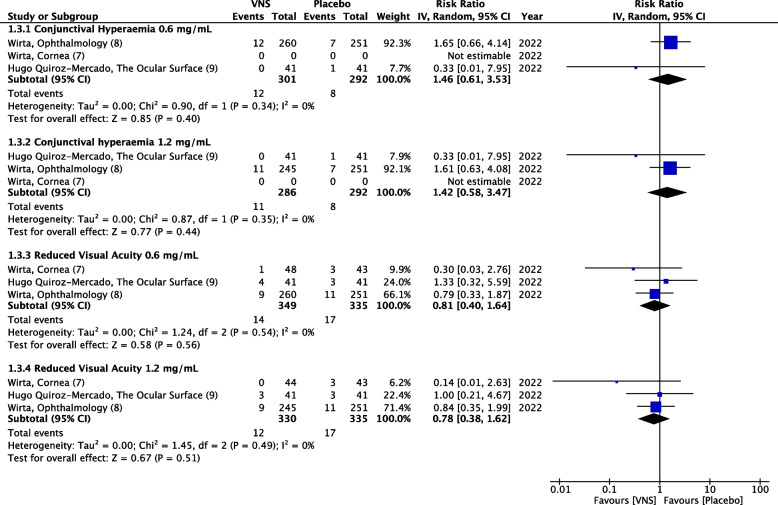
Forest plot of ocular adverse events. CI, confidence interval; IV, inverse variance; SD, standard deviation; VNS, Varenicline nasal spray

Conjunctival hyperemia was reported in two studies, while reduced visual acuity was reported in three RCTs. VNS did not cause any significant risk of conjunctival hyperemia in either mid-dose (RR = 1.46, 95% CI 0.61–3.53, *P* = 0.40, I^2^ = 0%) or high-dose (RR = 1.42, 95% CI 0.58–3.47, *P* = 0.44, I^2^ = 0%) (Fig. 7) (High quality of evidence) (Fig. 5). Similarly, the pooled estimate of events of reduced visual acuity showed no differences between VNS and placebo. Mid-dose (RR = 0.81, 95% CI 0.40–1.64, *P* = 0.56, I^2^ = 0%) and high-dose (RR = 0.78, 95% CI 0.38–1.62, *P* = 0.51, I^2^ = 0%) did not cause increased events of reduced visual acuity. (Fig. 7) (High quality of evidence) (Fig. 5). Both doses did not demonstrate a statistically significant difference in causing events of conjunctival hyperemia (*P* = 0.96, I^2^ = 0%) or reduced visual acuity (*P* = 0.94, I^2^ = 0%) (Fig. 7) (High quality of evidence) (Fig. 5).

### Nasal cavity-related adverse events

**Fig. 8:**
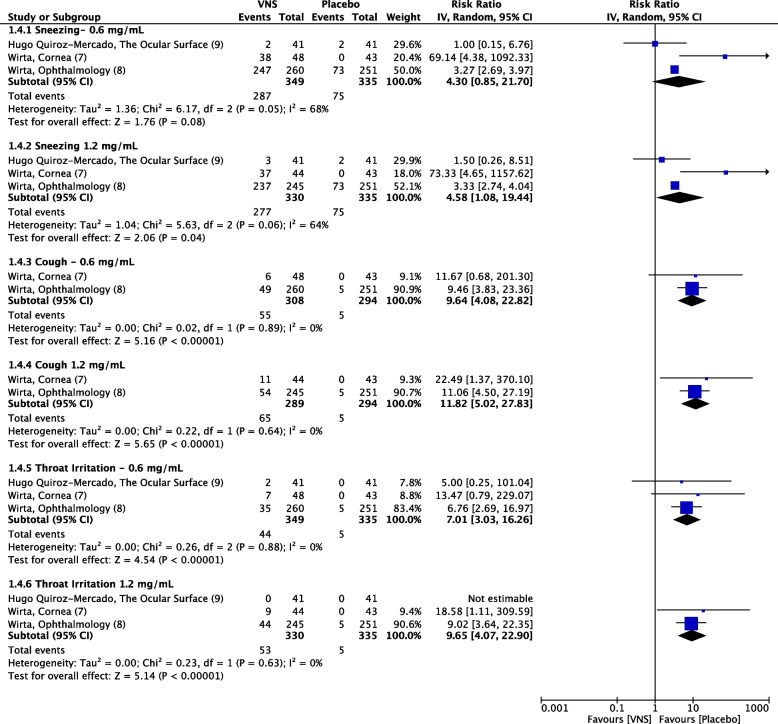
Forest plot of nasal cavity-related adverse events. CI, confidence interval; IV, inverse variance; SD, standard deviation; VNS, Varenicline nasal spray

Three RCTs reported adverse events of interest related to the nasal cavity. VNS caused slightly raised but insignificant events of sneezing both mid-dose (RR = 4.30, 95% CI 0.85–21.70, *P* = 0.08, I^2^ = 68%) and high-dose (RR = 4.58, 95% CI 1.08–19.44, *P* = 0.06, I^2^ = 64%) (Low quality of evidence) (Fig. 5). On the other hand, cough events had a significantly greater incidence in the VNS versus placebo. Mid-dose (RR = 9.64, 95% CI 4.08–22.82, *P* < 0.00001, I^2^ = 0%) and high-dose (RR = 11.82, 95% CI 5.02–27.83, *P* < 0.00001, I^2^ = 0%) (High quality of evidence) (Fig. 5). Similarly, mid-dose (RR = 7.01, 95% CI 3.03–16.26, *P* < 0.00001, I^2^ = 0%) and high-dose (RR = 9.65, 95% CI 4.07–22.90, *P* < 0.00001, I^2^ = 0%) of VNS caused throat irritation significantly (High quality of evidence) (Fig. 5). No dose-related differences were found between the incidence of sneezing (*P* = 0.95, I^2^ = 0%), cough (*P* = 0.74, I^2^ = 0%), and throat irritation (*P* = 0.60, I^2^ = 0%) (Fig. 8).

## Discussion

Our systematic review and meta-analysis evaluated the efficacy and safety of VNS for managing DED. VNS caused a significant improvement in DED measured with STS. VNS did not cause an increased incidence of SAEs and ocular AEs. On the other hand, the VNS arm caused a significant incidence of some nasal cavity-related AEs (cough and throat irritation only).

There are multiple options for the management of DED, ranging from artificial tear substitutes and anti-inflammatory eye drops to omega-3 fatty acids, hyaluronic acids, tetracyclines, and secretagogues [27–30]. Artificial tears are mainly the ophthalmologists’ first-line option in DED treatment. They are affordable and have been shown to improve the patient's quality of life by improving signs and symptoms and preventing DED aggravation. Nevertheless, they represent a significant burden to patients in terms of regular instillation and the noxious effects of their additive preservatives. Later, preservative-free tear supplementation was introduced to overcome these challenges but confronted other challenges, such as storage inconvenience and high cost, and was assessed to be non-superior to preservative-conjugated tear eye drop efficacy and safety in a recent systematic review [31, 32]. Steroidal anti-inflammatory topical applications were tested as alternative options and were effective but predisposed to glaucoma, cataracts, and other complications. Other anti-inflammatory applications of nonsteroidal derivatives were suggested to avoid these complications. Subsequently, the FDA approved cyclosporine as a treatment option for moderate to severe DED. A systematic review and meta-analysis in 2020 concluded that cyclosporine had substantial efficacy in treating DED, which was attenuated upon combining it with artificial tears [33]. A Cochrane review had low-certainty evidence on whether cyclosporine and artificial tears combination had a superior effect on reducing the symptoms and signs of DED compared to artificial tears monotherapy. Moreover, they found inconsistent low-certainty evidence of cyclosporine efficacy in providing a beneficial effect concerning tear production and stability [34].

VNS is a novel potential treatment for DED; however, it lacks sufficient evidence to establish or rule it out as a clinically feasible option for DED treatment. Hence, more high-quality evidence is needed. Thus, the comparison of our findings to previous results was not plausible. Our study is the first systematic review to assess the efficacy and safety of VNS for DED management.

To date, no gold standard has been established for DED diagnosis. One of the tests used for the detection of the quantity of basal and reflex tears is STS. STS is measured using filter paper applied to the lower eyelid, and the length of the wet part of the paper is measured in millimeters. It has been used for a lengthy time, and several ophthalmologists question its ability to measure DED signs due to significant fluctuation and lack of accuracy and reproducibility. Nevertheless, the FDA had listed anesthetized STS as one of the valid endpoints to establish the efficacy of interventions for DED [35–38].

The RCTs included in this study measured improvement in DED with either multiple time points, outcome tests, or both. In included RCTs, STS was reported at baseline and on the 28^th^ and 84^th^ day. However, the 28^th^ day was a common time point across the included studies. This specific time point was used in the meta-analysis to ensure the precision and comparability of pooled results. Our meta-analysis showed high I^2^ in the efficacy outcome, indicating a high level of heterogeneity; nonetheless, both individual and pooled effects favored the use of VNS versus placebo. In general, explanations for heterogeneity, other than clinical variations, might include methodological concerns such as problems with randomization, early termination of trials, and publication bias [39]. In the present study, the high and statistically significant heterogeneity was not clearly understood, yet this may be partially explained by the fact that one of the included trials (Quiroz-Mercado et al.) was a single-centered study. A critical limitation of the single-subject research design is the generalisability of the study conclusions. Additionally, Quiroz-Mercado et al. trial investigated a specific ethnic group (i.e., almost all the study participants were Hispanic). Although it may be a strength as it adds to a higher population of Hispanic patients in the assessment of safety and efficacy as compared to other DED clinical trials to support approved medications, such selection may add to the heterogeneity as the other included trials were multi-centered and not favoring or including most of their participants from a specific ethnic group. All these explanations and assumptions are made to determine the reason for the high heterogeneity. Leave-one-out sensitivity analysis of all included studies was done to investigate which study is causing the heterogeneity. Sensitivity analysis did not reduce substantial heterogeneity by excluding each study individually from the pooled estimate of the efficacy outcome. Trial protocols of included RCTs state that an adverse event is considered "serious" if, in the evaluator's view, it results in death, a life-threatening event, inpatient hospitalization, prolongation of existing hospitalization, an incapacitating or substantial disruption of the ability to perform a day-to-day activity, or a congenital anomaly in an offspring of a study participant. With such a relatively expansive definition of SAEs, tens of events were reported that, most likely, had a minor or no logical relation to the administration of VNS. AEs, on the other hand, were defined as side effects that do not fit with SAEs’ definition or cause mortality [7–9]. Quiroz-Mercado et al. was the only study not to report the incidence of any SAEs in both doses, while Wirta et al., Cornea, did not report an SAE in the high-dose group only. This might be attributed to the low sample size of both studies [7, 9]. SAEs that were reported included atrial fibrillation, coronavirus infection, sepsis, lung neoplasm, pneumonia, intervertebral disc protrusion, hypertensive urgency, and atrioventricular block in the placebo arm. In addition, bradycardia, coronary artery disease, myocardial infarction, Arnold-Chiari malformation, umbilical hernia, acute cholecystitis, sepsis, diabetic gangrene, osteomyelitis, and others occurred in the VNS group. In contrast, VNS was affiliated with qualitatively relevant AEs. Conjunctival hyperemia and reduced visual acuity were the most reported AEs, occasionally reaching a several-fold increase in the incidence of the VNS group versus placebo. Side effects of VNS mostly related to the nasal cavity, such as sneezing, due to its ROA and MOA. Nasal sprays, even as vehicle sprays, cause a reflex response that is mediated by triggering the TPP in the nasal cavity. This, in addition to the MOA of VNS as a nAChR agonist, causes an increased incidence of transient sneezing (lasting less than two minutes) after nasal spray administration [12]. This might explain the slightly larger effect size of sneezing events in the high-dose (SMD = 4.58) versus the mid-dose (SMD = 4.30) though both were similar to vehicle nasal spray, respectively (*P* = 0.06) (*P* = 0.08). Sneezing was the most common nasal cavity-related AE by a significant margin, followed by cough and throat irritation [7–9]. At pilot meta-analysis, a pooled estimate of all nonserious AEs was done as per protocol, but this resulted in vague conclusions about the quality of these AEs. Later, a meta-analysis of the most common AEs of ocular and nasal cavity origin was conducted. Additionally, the subgroup analysis in this study compared mid-dose and high doses of VNS, yet no significant differences were found between them in safety or efficacy outcomes. A lower dose was tested in Wirta et al., Cornea, with a 0.12 mg/mL concentration, but it was not assessed in other RCTs, which made it incomparable to other doses in the meta-analysis [7]. Thus, the mid-dose is the lowest effective dose, according to our meta-analysis.

As VNS is a novel treatment, more RCTs are needed to evaluate its viability for DED management, and more diverse participant characteristics, such as sex and ethnicity, should be considered. This article is the first approach to systematically evaluate, appraise, and analyze this topic and perform subgroup analysis of different doses. All included RCTs had a low risk of bias in the Cochrane risk of bias assessment. Funnel plots were not used in this study due to the low number of included studies, which made visual detection of publication bias not feasible.

This study has several limitations. One of which is the low number of RCTs due to the novelty of the intervention. Usually, the high inconsistency of pooled results may be attributed to methodological differences that led to high I^2^, which is concluded as high heterogeneity. However, from the authors' point of view, these high heterogeneity levels are not a limitation in our meta-analysis since a proper investigation of its cause was done in addition to the fact that all included RCTs in our paper were conducted by the pharmaceutical company manufacturing VNS and had nearly identical RCT protocols and procedures.

## Conclusions

Overall, VNS is an effective treatment for DED. It causes a significant improvement in tear production and DED signs and is superior to vehicle nasal spray. Nonetheless, AEs should be considered, as VNS causes a higher incidence of cough and throat irritation. However, it does not cause sneezing, SAEs, or ocular AEs. No differences were detected between doses in any of the measured outcomes. More seamlessly structured RCTs are needed to study different doses and interventions with similar MOA or ROA. Upcoming studies should also compare its safety and efficacy against currently established management options for DED.

## Supplementary Information

**Additional file 1.**

## Data Availability

The search strategy and results are attached in the supplementary material. Raw data of RCTs included in this study are available through their corresponding references (7–9). Further data are available upon reasonable request from the corresponding author (Bader Bashrahil).

## Abbreviations

DED: Dry eye disease
VNS: Varenicline nasal spray
FDA: The United States Food and Drug Authority
GRADE: Grading of Recommendations Assessment, Development, and Evaluation
CENTRAL: Cochrane Central Register of Controlled Trials
RCTs: Randomized controlled trial
SAEs: Serious adverse events
AEs: Adverse events
SMD: Standardized mean difference
RR: Risk Ratio
MOA: Mechanism of action
ROA: Route of administration
