# Supplementary material for: The efficacy and safety of varenicline nasal spray for the management of dry eye signs: a systematic review and meta-analysis
Source: BMC Ophthalmol. 2023 Jul 14;23:319. doi: 10.1186/s12886-023-03069-y (PMC10347795; doi:10.1186/s12886-023-03069-y)
Supplement: Supplementary file 1 — Additional file 1. [file 12886_2023_3069_MOESM1_ESM.docx]

Dry eye OR Dry eye disease OR Dry eye syndrome OR Keratoconjunctivitis Sicca

Varenicline OR Nasal Spray OR OC-01

Randomized Controlled Trial OR RCT OR Clinical Trial OR Trial

**Search history: 06/07/2022**

ID Search

#1 MeSH descriptor: [Dry Eye Syndromes] explode all trees

#2 MeSH descriptor: [Keratoconjunctivitis Sicca] explode all trees

#3 ("dry eye"):ti,ab,kw OR (Dry eye disease):ti,ab,kw OR ("dry eye syndrome"):ti,ab,kw OR ("keratoconjunctivitis sicca"):ti,ab,kw (Word variations have been searched)

#4 #1 OR #2 OR #3

#5 MeSH descriptor: [Varenicline] explode all trees

#6 (OC-01):ti,ab,kw OR ("varenicline"):ti,ab,kw OR (OC-01):ti,ab,kw (Word variations have been searched)

#7 #5 OR #6

#8 MeSH descriptor: [Randomized Controlled Trial] explode all trees

#9 MeSH descriptor: [Clinical Trial] explode all trees

#10 ("randomized controlled trial"):ti,ab,kw OR (RCT):ti,ab,kw OR ("clinical trial"):ti,ab,kw OR (Trial):ti,ab,kw (Word variations have been searched)

#11 #8 OR #9 OR #10

#12 #4 AND #7 AND #11

CENTRAL=11

EMBASE=6

MEDLINE=8

| **Search number** | **Query** | **Sort By** | **Filters** | **Search Details** | **Results** | **Time** |
| --- | --- | --- | --- | --- | --- | --- |
| **4** | #1 AND #2 AND #3 |  |  | ("dry eye syndromes"[MeSH Terms] OR ("dry"[All Fields] AND "eye"[All Fields] AND "syndromes"[All Fields]) OR "dry eye syndromes"[All Fields] OR ("dry"[All Fields] AND "eye"[All Fields]) OR "dry eye"[All Fields] OR "dry eye syndromes"[MeSH Terms] OR "dry eye syndromes"[MeSH Terms] OR ("dry eye syndromes"[MeSH Terms] OR ("dry"[All Fields] AND "eye"[All Fields] AND "syndromes"[All Fields]) OR "dry eye syndromes"[All Fields] OR ("dry"[All Fields] AND "eye"[All Fields] AND "disease"[All Fields]) OR "dry eye disease"[All Fields]) OR ("dry eye syndromes"[MeSH Terms] OR ("dry"[All Fields] AND "eye"[All Fields] AND "syndromes"[All Fields]) OR "dry eye syndromes"[All Fields] OR ("dry"[All Fields] AND "eye"[All Fields] AND "syndrome"[All Fields]) OR "dry eye syndrome"[All Fields]) OR "dry eye syndromes"[MeSH Terms] OR "keratoconjunctivitis sicca"[MeSH Terms] OR ("keratoconjunctivitis sicca"[MeSH Terms] OR ("keratoconjunctivitis"[All Fields] AND "sicca"[All Fields]) OR "keratoconjunctivitis sicca"[All Fields])) AND ("vareniclin"[All Fields] OR "varenicline"[MeSH Terms] OR "varenicline"[All Fields] OR "varenicline s"[All Fields] OR "varenicline"[MeSH Terms] OR "nasal sprays"[MeSH Terms] OR ("nasal sprays"[MeSH Terms] OR ("nasal"[All Fields] AND "sprays"[All Fields]) OR "nasal sprays"[All Fields] OR ("nasal"[All Fields] AND "spray"[All Fields]) OR "nasal spray"[All Fields]) OR "OC-01"[All Fields]) AND ("randomized controlled trial"[Publication Type] OR "randomized controlled trials as topic"[MeSH Terms] OR "randomized controlled trial"[All Fields] OR "randomised controlled trial"[All Fields] OR "randomized controlled trials as topic"[MeSH Terms] OR "RCT"[All Fields] OR ("clinical trial"[Publication Type] OR "clinical trials as topic"[MeSH Terms] OR "clinical trial"[All Fields]) OR "clinical trials as topic"[MeSH Terms] OR "clinical trials as topic"[MeSH Terms] OR ("clinical trials as topic"[MeSH Terms] OR ("clinical"[All Fields] AND "trials"[All Fields] AND "topic"[All Fields]) OR "clinical trials as topic"[All Fields] OR "trial"[All Fields] OR "trial s"[All Fields] OR "trialed"[All Fields] OR "trialing"[All Fields] OR "trials"[All Fields])) | 8 | 18:03:36 |
| **3** | (((((((Randomized Controlled Trial) OR (Randomized Controlled Trial[MeSH Terms])) OR (RCT[MeSH Terms])) OR (RCT)) OR (Clinical Trial)) OR (clinical trial[MeSH Terms])) OR (Trial[MeSH Terms])) OR (Trial) |  |  | "randomized controlled trial"[Publication Type] OR "randomized controlled trials as topic"[MeSH Terms] OR "randomized controlled trial"[All Fields] OR "randomised controlled trial"[All Fields] OR "randomized controlled trials as topic"[MeSH Terms] OR "RCT"[All Fields] OR ("clinical trial"[Publication Type] OR "clinical trials as topic"[MeSH Terms] OR "clinical trial"[All Fields]) OR "clinical trials as topic"[MeSH Terms] OR "clinical trials as topic"[MeSH Terms] OR ("clinical trials as topic"[MeSH Terms] OR ("clinical"[All Fields] AND "trials"[All Fields] AND "topic"[All Fields]) OR "clinical trials as topic"[All Fields] OR "trial"[All Fields] OR "trial s"[All Fields] OR "trialed"[All Fields] OR "trialing"[All Fields] OR "trials"[All Fields]) | 1,964,261 | 18:03:09 |
| **2** | (((((Varenicline) OR (Varenicline[MeSH Terms])) OR (nasal sprays[MeSH Terms])) OR (Nasal Spray)) OR (OC-01)) OR (OC-01[MeSH Terms]) |  |  | "vareniclin"[All Fields] OR "varenicline"[MeSH Terms] OR "varenicline"[All Fields] OR "varenicline s"[All Fields] OR "varenicline"[MeSH Terms] OR "nasal sprays"[MeSH Terms] OR ("nasal sprays"[MeSH Terms] OR ("nasal"[All Fields] AND "sprays"[All Fields]) OR "nasal sprays"[All Fields] OR ("nasal"[All Fields] AND "spray"[All Fields]) OR "nasal spray"[All Fields]) OR "OC-01"[All Fields] | 6,759 | 18:02:08 |
| **1** | (((((((Dry eye) OR (Dry eye[MeSH Terms])) OR (Dry eye disease[MeSH Terms])) OR (Dry eye disease)) OR (Dry eye syndrome)) OR (Dry eye syndrome[MeSH Terms])) OR (Keratoconjunctivitis Sicca[MeSH Terms])) OR (Keratoconjunctivitis Sicca) |  |  | "dry eye syndromes"[MeSH Terms] OR ("dry"[All Fields] AND "eye"[All Fields] AND "syndromes"[All Fields]) OR "dry eye syndromes"[All Fields] OR ("dry"[All Fields] AND "eye"[All Fields]) OR "dry eye"[All Fields] OR "dry eye syndromes"[MeSH Terms] OR "dry eye syndromes"[MeSH Terms] OR ("dry eye syndromes"[MeSH Terms] OR ("dry"[All Fields] AND "eye"[All Fields] AND "syndromes"[All Fields]) OR "dry eye syndromes"[All Fields] OR ("dry"[All Fields] AND "eye"[All Fields] AND "disease"[All Fields]) OR "dry eye disease"[All Fields]) OR ("dry eye syndromes"[MeSH Terms] OR ("dry"[All Fields] AND "eye"[All Fields] AND "syndromes"[All Fields]) OR "dry eye syndromes"[All Fields] OR ("dry"[All Fields] AND "eye"[All Fields] AND "syndrome"[All Fields]) OR "dry eye syndrome"[All Fields]) OR "dry eye syndromes"[MeSH Terms] OR "keratoconjunctivitis sicca"[MeSH Terms] OR ("keratoconjunctivitis sicca"[MeSH Terms] OR ("keratoconjunctivitis"[All Fields] AND "sicca"[All Fields]) OR "keratoconjunctivitis sicca"[All Fields]) | 27,763 | 18:01:17 |
